# World Health Day focus on HIV and depression – a comorbidity with specific challenges

**DOI:** 10.7448/IAS.20.1.21956

**Published:** 2017-04-07

**Authors:** Lorraine Sherr, Lucie Cluver

**Affiliations:** ^a^ Research Department of Infection and Population Health, University College London, London, UK; ^b^ Centre for Evidence Based Intervention, Department of Social Policy and Intervention, Oxford University, Oxford, UK

Depression is intricately bound with HIV [1]. Depression may elevate risks of HIV acquisition [2] and be triggered by an HIV diagnosis [3], in people living with HIV [4] or in people receiving treatment [5,6]. Depression is a key comorbidity in HIV [7]. Mental health is often underserved especially in adult care, and more so for children and adolescents – despite the elevated infection rates [8], non-adherence and mortality in these age groups.

Pregnant HIV-positive women experience both pre- and postnatal depression [9,10], which may affect quality of caring, feeding [11], stimulation and child development [12]. Interventions are available to treat both maternal and paternal depression [13–16] but need to be integrated into care. Children themselves can experience HIV-associated depression and mood-related disorders, as high as 63% for affected vs. 7% for non-affected comparison groups in Ghana [17] with similarly elevated rates in South Africa, Uganda and Rwanda [18–20]. Just as with adults, childhood depression can be reactive – to family ill health, bereavement, changes in care arrangements, an HIV diagnosis or a death [21]. Mood is also sensitive to social factors, and studies in China show direct relationships of AIDS-related stigma to child depression [22]. Depression is predicted by gender, education, bereavement, disclosure, bullying [23], and caregiving environments [24,25]. Depression in children is associated with difficulties in coping, adaptation, risk behaviours and non-adherence [26], presenting a major risk to survival and well-being [27,28].

Interventions are available [29], including community-based organization support [30,31], home visiting [32], lay support [33], social support [34], quality caregiving [35], technology-delivered interventions [36] and management programmes [37]. However, much of the response is contained with lay providers and if quality mental health services are to be available, government and policy providers need to pay attention to training, infrastructure, integration and quality provision. Mental health provision is the next test [38].
